# Antiproliferative effects of sulphonamide carbonic anhydrase inhibitors C18, SLC-0111 and acetazolamide on bladder, glioblastoma and pancreatic cancer cell lines

**DOI:** 10.1080/14756366.2021.2004592

**Published:** 2021-12-11

**Authors:** Silvia Mussi, Sara Rezzola, Paola Chiodelli, Alessio Nocentini, Claudiu T. Supuran, Roberto Ronca

**Affiliations:** a Experimental Oncology and Immunology, Department of Molecular and Translational Medicine, University of Brescia, Brescia, Italy; b NEUROFARBA Department, Sezione di Scienze Farmaceutiche e Nutraceutiche, University of Florence, Sesto Fiorentino, Florence, Italy

**Keywords:** Carbonic anhydrase inhibitors, glioblastoma, bladder cancer, pancreatic cancer, SLC-0111

## Abstract

Carbonic anhydrase IX/XII (CA IX/XII), are cell-surface enzymes typically expressed by cancer cells as a form of adaptation to hypoxia and acidosis. It has been widely reported that these proteins play pivotal roles in cancer progression fostering cell migration, aggressiveness and resistance to first line chemo- and radiotherapies. CA IX has emerged as a promising target in cancer therapy and several approaches and families of compounds were characterised in the attempt to find optimal targeting by inhibiting of the high catalytic activity of the enzyme. In the present work, different cell lines representing glioblastoma, bladder and pancreatic cancer have been exploited to compare the inhibitory and antiproliferative effect of primary sulphonamide acetazolamide (AAZ), the Phase Ib/II clinical grade sulphonamide SLC-0111, and a membrane-impermeant positively charged, pyridinium-derivative (C18). New hints regarding the possibility to exploit CA inhibitors in these cancer types are proposed.

## 1. Introduction

In the tumour contexts, the rapid and uncontrolled proliferation of neoplastic cells is mainly limited by the availability of oxygen, due to the insufficiency of blood supply. Hypoxia thus represents a typical feature of the majority of solid tumours. It has been reported that chronic (but also cycling) hypoxia leads to a range of diverse phenomena, among which increased formation of reactive oxygen species (ROS) contributing to increased genetic instability and mutagenesis^1^^,^^2^, that leads to tumour cell survival and progression^3^. In addition, the hypoxic milieu has been associated with increased resistance to the metastatic phenotype and resistance to radiation therapy and chemotherapy^4–8^. Due to the high proliferative rate and metabolic turnover, cancer cells exploit the oxidative phosphorylation of glucose and its anaerobic glycolysis for energy production, thus generating high quantities of metabolic acids that require the intervention of a various protein complexes involved in the prevention of intracellular accumulation of H^+^ ions, and the maintenance of an intracellular alkaline pH^3^^,^^4^. In this context, the hypoxia-inducible factor-1 (HIF-1) represents the main transcription factor activated in response to hypoxia and driving the regulations of many cellular biological processes including glycolysis, cell migration, angiogenesis, and pH regulation^1–4^. In particular, HIF-1 can regulate the expression and activation of various pH-regulating proteins, such as sodium bicarbonate transporters, sodium-proton exchangers, monocarboxylate transporter-4 (MCT-4), and the carbonic anhydrase IX/XII (CA IX/XII) isoforms, that lead to an inverted pH gradient, typical of cancer cells^9^, i.e. acidic extracellular pH and slightly more alkaline cytosolic pH values compared to normal ones.

CA IX and XII are cell-surface enzymes overexpressed in hypoxia, belonging to the α-CA (EC 4.2.1.1) family of zinc metalloenzymes, and catalyse the reversible hydration of CO_2_ to bicarbonate ions (HCO_3_^−^) and protons (H^+^)^10^. These enzymes are involved in the adaptation of cancer cells to acidosis, but are also involved in ferroptosis and several other processes by which tumour cells escape control and are able to proliferate^10^. High levels of CA IX/XII have been reported in several types of cancer where it promotes cell survival under hypoxic conditions and may favour tumour cells migration and aggressiveness^11–13^. Moreover, the expression of CA IX has been correlated with poor prognosis in breast, ovarian, pancreatic, bladder, brain and other human tumours^14^^,^^15^, and its targeting has been widely proposed as a therapeutic approach to treat aggressive cancers^16^. To this purpose different inhibitors have been developed and characterised acting as blockers of the CA IX activity^3^^,^^4^, including the sulphonamides, sulphonamide derivatives, other small molecules inhibitors, and antibody/cytokine-drug conjugates^17^^,^^18^. In the present work we compare the *in vitro* effect of different CA IX inhibitors on various tumour types. In particular, we compare the biological activity of the primary sulphonamide acetazolamide (AAZ)^18^, the sulphonamide SLC-0111, actually in Phase Ib/II clinical trials for the management of advanced solid tumours^19^, and a positively charged, pyridinium-derivative (C18) characterised by the fact that it is membrane impermeant^20^.

## 2. Materials and methods

### 2.1. Cell culture and reagents

Human bladder cancer RT4 cells were obtained from ATCC-LGC Standards Repository (ATCC number HTB-2) and maintained in McCoy’s 5 A medium supplemented with 10% heat-inactivated FCS. Human bladder cancer 5637 cells (ATCC HTB-9), Human pancreatic cancer CF-PAC-1 (ATCC CRL-1918) and PANC-1 (ATCC CRL-1469) cells were grown in RPMI 1640 supplemented with 10% FCS. Human bladder cancer HT-137 cells (ATCC CRL-1472), human glioblastoma U87MG (ATCC HTB-14), U251 (Merck U-251 MG), T98G (ATCC CRL-1690) cells were grown in DMEM supplemented with 10% FCS. Cells were kept at low passage, returning to original frozen stocks every 3 to 4 months. Hypoxic culture conditions were realised in the presence of 1% O_2_ e 5% CO_2_. AAZ (acetazolamide) was commercially available from Sigma-Aldrich (Milan, Italy), whereas SLC-0111^21^ and C18^20^ were prepared as reported earlier^20^^,^^21^.

### 2.2. Western blot analysis

Cells were cultured at 37 °C with 1% O_2_, 5% CO_2_ for 24 h and lysed in lysis buffer (TRIS-HCl pH 7 50 mM, NaCl 150 mM, Triton X-100 1%, BriJ 0.1%). Protein concentrations were determined using the Bradford protein assay (Bio-Rad Laboratories, Milano, Italy). Then, 60 μg protein/sample were separated by SDS-PAGE, analysed by WB for CA9 with the murine mAb M75 an IgG2b and normalised with an anti-GAPDH (Santa Cruz Biotechnology).

### 2.3. Cell proliferation assay

The different cellular lines were seeded in 48-well plates and treated in 1% FBS with increasing concentrations of AAZ, SLC-001 or C18. After 72 h of incubation at 37 °C with 1% O_2_, 5% CO_2_, cells were trypsinized and cell counting was performed with the MACSQuant^®^ Analyzer (Miltenyi Biotec).

## 3. Results and discussion

### 3.1. Ca inhibitory effects of sulphonamides C18, SLC-0111 and acetazolamide

Sulphonamides constitute one of the most investigated class of CA inhibitors (CAIs), with many such compounds on clinical use for decades for the management of many diseases connected with CA imbalances, or more recently, in clinical trials as antitumor agents^16^^,^^17^.

**Figure 1.**
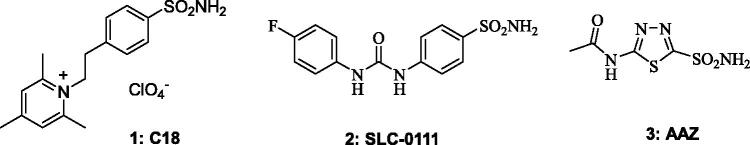
Sulphonamides **1–3** investigated in this work, **C18**, **SLC-0111** and **AAZ**.

We decided to investigate here three such CAIs with very different properties and CA inhibition profiles against the two human (h) hCA isoforms involved in tumorigenesis (Figure 1 and Table 1), hCA IX and XII, as well as the two main off-target isoforms, the cytosolic hCA I and II^3^^,^^16^^,^^17^. Indeed, compound **1**, also known as C18, belongs to a class of positively-charged, membrane-impermeant compounds^23^ that were demonstrated to be unable to cross plasma membranes and thus selectively inhibit transmembrane isoforms such as CA IX and XII^23^^,^^24^. C18 is an effective, low nanomolar inhibitor of CA II, IX and XII, being a weak inhibitor against hCA I (see Table 1 and^16^) However, due to its membrane-impermeability, *in vivo* it should predominantly inhibit the transmembrane isoforms, such as CA IX and XII^23^. SLC-0111, compound **2**, as mentioned above, has been designed by the tail approach as a selective CAI for the tumour-associated isoforms^21^. The compound is a low nanomolar CA IX/XII inhibitor, whereas its activity against the off-target isoforms hCA I and II is in the micromolar range (Table 1 and^21^) Acetazolamide **3**, is the CAI *par excellence*, being in clinical use for almost 70 years as a diuretic, antiglaucoma and antiepileptic agent^16^, being also employed for the management of other conditions such as mountain sickness, idiopathic intracranial hypertension, and other CNS or renal conditions^16^^,^^17^^,^^25^. As seen from data of Table 1, AAZ is a promiscuous, highly effective CAI against all the 4 isoforms considered here, CA I, II, IX and XII.

**Table 1.** CA inhibition data with sulphonamides **1–3** against human CA isoforms of clinical relevance.

	K_I_ (nM)*			
Compound	hCA I	hCA II	hCA IX	hCA XII
**1** ^16^	4000	21	14	7
**2** ^21^	5080	960	4.5	45
**3** ^16^	250	12	25	5.7

*The reported K_I_-s are from references^16^^,^^21^ and were obtained by a stopped-flow, CO_2_ hydrase assay^22^ using purified recombinant CA isoforms.

### 3.2. Evaluation of sulphonamide CAIs effects on bladder cancer cells

**Figure 2.**
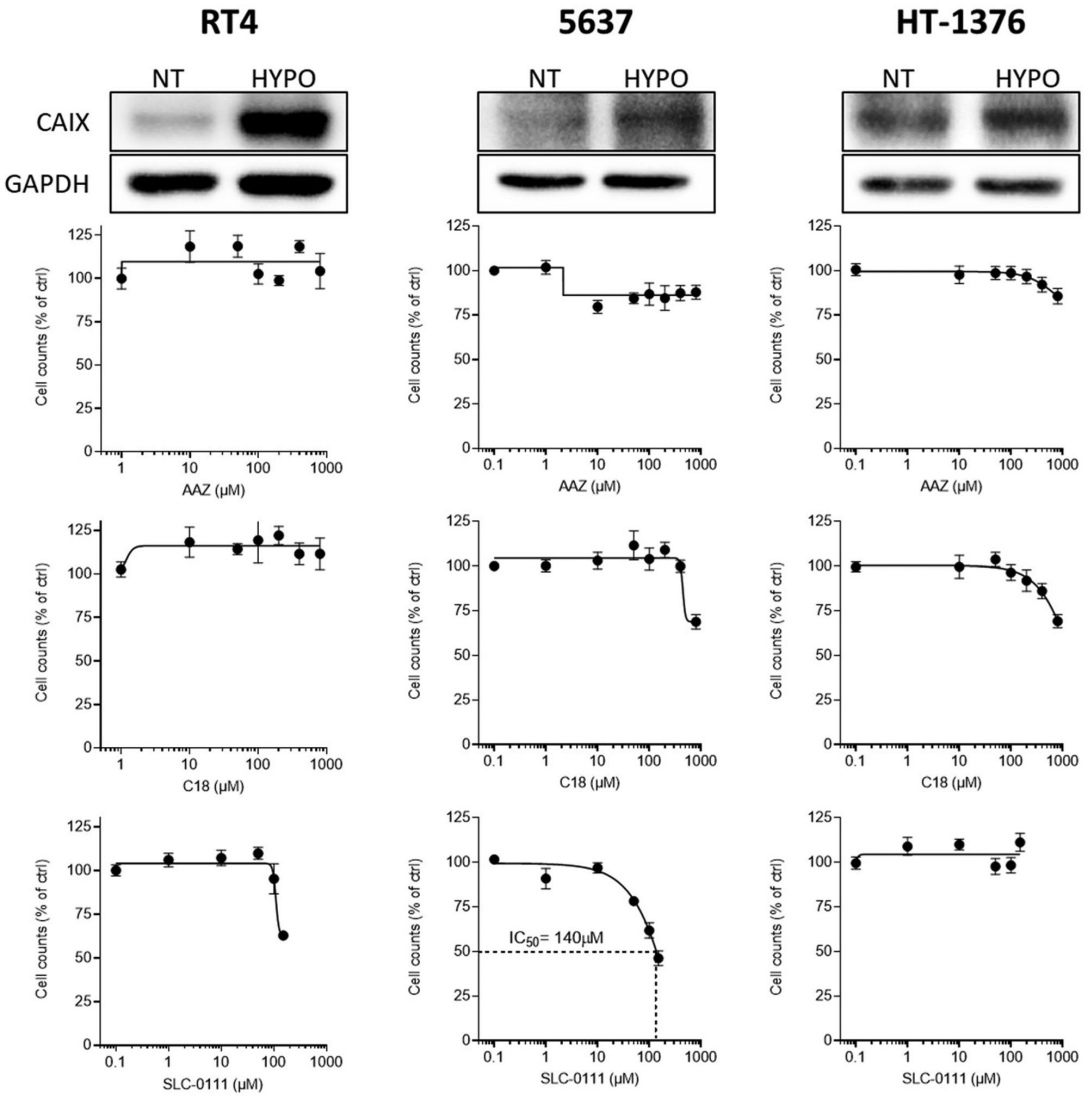
Protein levels of CA IX in bladder cancer cells under normal (NT) or hypoxic (HYPO) conditions (top panels), and cell proliferation of these cells treated with **C18**, **SLC-0111** or **AAZ**.

We evaluated the effect of AAZ, SLC-0111 and C18 on three tumour cell lines representing different grades of bladder cancer progression: low grade/papilloma-like RT4 cells, grade II 5637 cells, and grade III/muscle invasive HT-1376 cells. Firstly, we analysed CAIX expression under hypoxic conditions. As shown in Figure 2, all the bladder cancer cells express basal levels of CAIX that significantly rise after exposure to hypoxic conditions for 24 h. We compared the efficacy of the three CAIX inhibitors to inhibit the proliferation of these after 72 h of treatment in hypoxia. As shown in Figure 2, both the AAZ had no effect on cell proliferation on all cell lines. Similarly, the membrane impermeant C18 compound had a very mild effect on proliferation of medium (5637) and high-grade cells (HT-1376), but only at high concentrations. Finally, the clinical grade SLC-0111 drug displayed a modest inhibitory effect on RT4 and 5637 cells, but not on HT-1376 cells.

These data suggest that bladder cancer cells, regardless from their grade and aggressiveness are not particularly sensitive to the inhibition of CA IX.

### 3.3. Evaluation of sulphonamide CAI effects on glioblastoma cells

**Figure 3.**
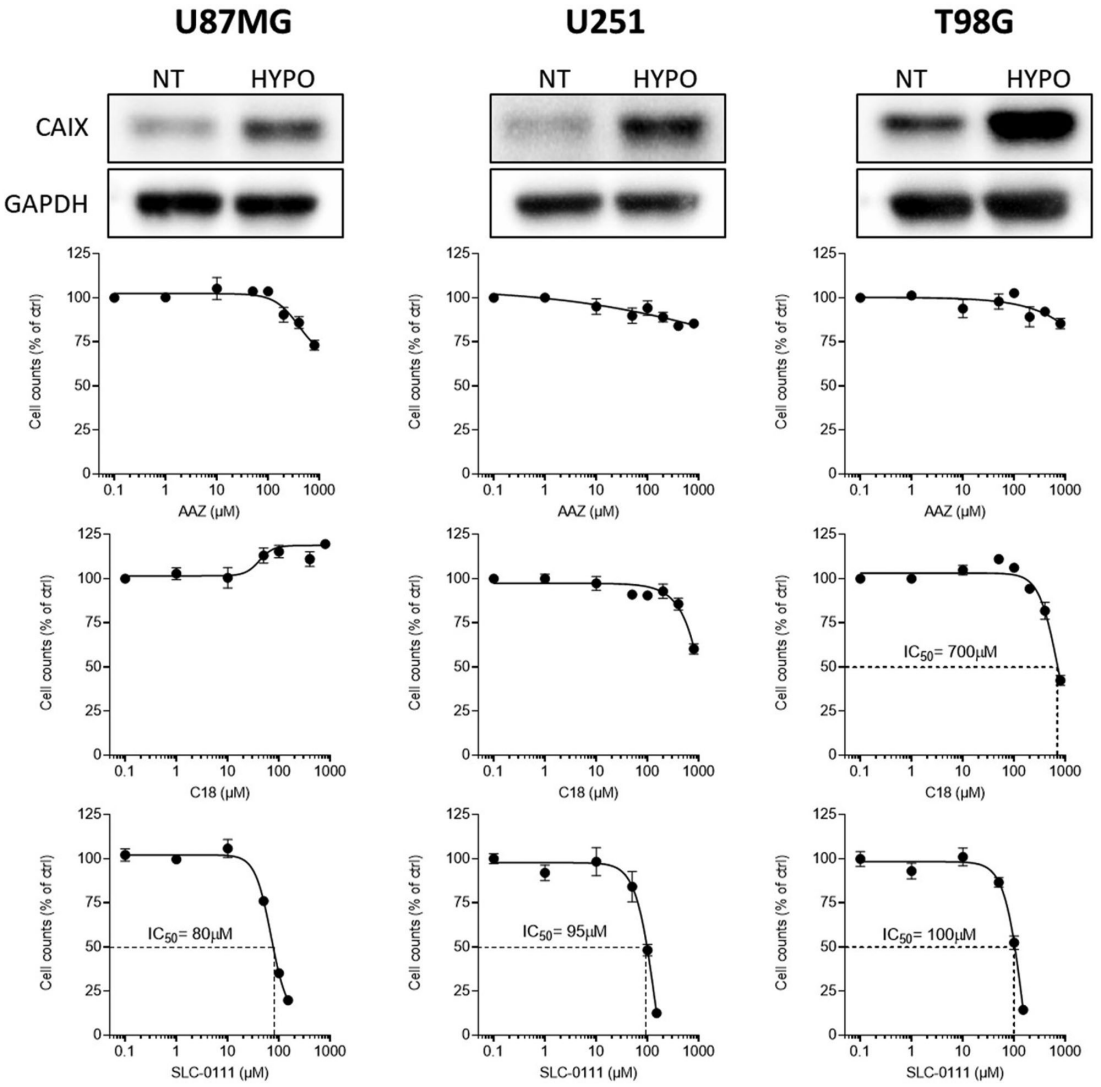
Protein levels of CA IX in glioblastoma cells under normal (NT) or hypoxic (HYPO) conditions (top panels), and cell proliferation of these cells treated with **C18**, **SLC-0111** or **AAZ**.

The expression of CAIX was evaluated by Western blot in three glioblastoma cell lines maintained for 24 h in hypoxic conditions. As shown in Figure 3, CA IX levels are significantly increased after exposure to hypoxia for 24 h. We then evaluated the anti-proliferative effect of the three CA IX inhibitors under these conditions. No effect was observed when cells were treated with AAZ for 72 h, while a mild effect was observed after treatment with C18 in U251 and in T98G cells. Notably, the clinical grade SLC-0111 compound showed a significant effect on all the three cell lines, with an IC_50_ ranging from 80 to 100 µM. These data suggest that CA IX blockade can significantly impact on glioblastoma cells, and that the loss of impermeant C18 drug has a mild efficacy in comparison with SLC-0111, but a better profile in respect to AAZ.

### 3.4. Evaluation of sulphonamides effects on pancreatic cancer cells

**Figure 4.**
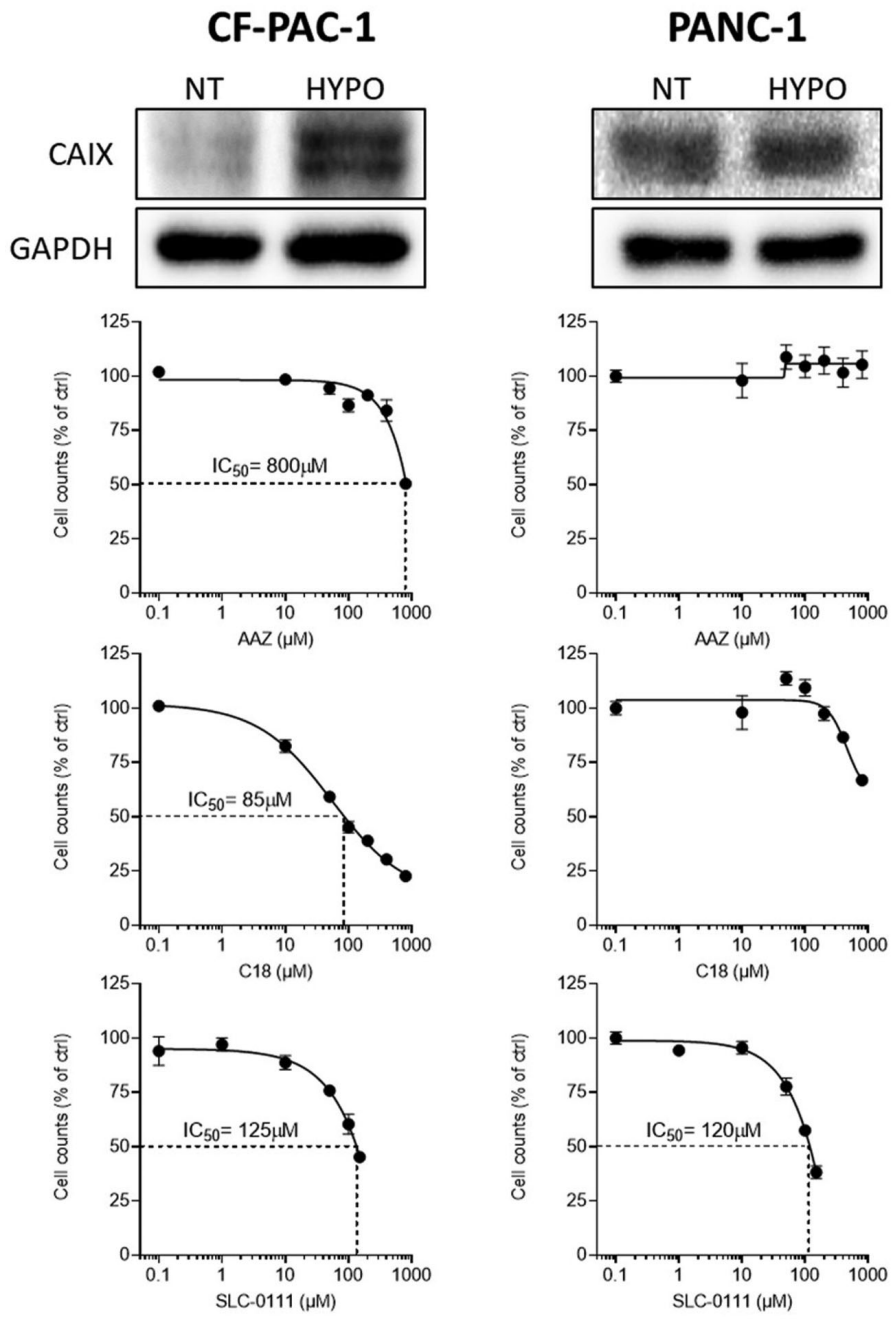
Protein levels of CA IX in pancreatic cancer cells under normal (NT) or hypoxic (HYPO) conditions (top panels), and cell proliferation of these cells treated with **C18**, **SLC-0111** or **AAZ**.

In line with our previous experiments, we confirmed the expression of CA IX in two cell lines of pancreatic cancer (CF-PAC-1 and PANC-1 cells) under hypoxic conditions (Figure 4). As shown in Figure 4 we treatment with AAZ slightly inhibited the proliferation only in CF-PAC-1 cells with no effect on the proliferation of PANC-1 cells even at very high concentrations. Interestingly, the membrane impermeant C18 compound was able to reduce cell proliferation in CF-PAC-1 cells, with an IC_50_≈85 µM and a very modest effect on PANC-1 cells. Finally, SLC-0111 impaired cell proliferation in both pancreatic cell lines with an IC_50_ ≈120–125 µM. These data point to a promising effect of CA IX inhibition in pancreatic cancer cells and show a slight effect of C18 compound in respect to what observed in other cancer models.

## 4. Conclusions

Sulphonamide CAIs started to be investigated as potential anti-tumour agents in the last two decades after the discovery of the two tumour-associated isoforms CA IX and XII, and their upregulation in hypoxia^3^^,^^4^. Nowadays, a large number of such derivatives were characterised in detail regarding their *in vitro* inhibitory profile against the target and off-target isoforms, but also considering pharmacokinetic and pharmacodynamics properties^26^. In fact, although many low nanomolar *in vitro* CAIs were reported, in cellular systems or *in vivo*, in animal models, not all these compounds showed the expected anti-proliferative activity^26^. Although such effects are reported for most classes of antitumor agents, here we evaluated three sulphonamide CAIs with very different inhibition profiles and physico-chemical properties in order to possibly address this conundrum: a positively-charged, membrane-impermeant compound (C18) with a rather selective CA IX/XII inhibition; the drug candidate in Phase Ib/II clinical trials SLC-0111 which is a CA IX/XII-selective inhibitor, and the pan-inhibitor acetazolamide. All these three compounds are highly effective against the target enzymes CA IX and XII, but they possess variable activity against the off-target isoforms (Table 1). The data obtained against several tumour cell lines expressing variable amounts of CA IX reported here, indeed show a quite different antiproliferative activity of the three compounds. For instance, bladder cancer cells representing different grade of this urological neoplasia are mostly insensitive to all CAIs tested, apart from SLC-0111 that showed only a modest effect in the medium grade invasive 5637 cells. Interestingly, in the more aggressive and invasive cancer types, represented by glioblastoma and pancreatic cancer cells, the clinical candidate SLC-0111 exerted a significant antiproliferative effect. These results were confirmed in all the five cell lines tested and suggest that in these highly proliferating cancers the inhibition of CA IX might provide a beneficial therapeutic effect. In addition, when the membrane impermeant compound C18 was tested in these tumour cells, its activity was generally poor in glioblastoma cells, but a promising activity was observed in one of the pancreatic cancer lines.

In general, our data suggest that more aggressive tumours might be more sensitive to CA IX inhibition and this should be taken into consideration in addition to the simple presence/expression of the target. Indeed, even though *in vivo* validation of these observations will be required, highly proliferative tumours are expected to be more prone to generate a hypoxic environment and thus, be more sensitive to CA blockers. As shown here, the effect and role of CA IX has been reported in different tumour settings and, even if the *in vivo* data clearly reveal the key role of these proteins in tumour progression, resistance to therapy and promotion of tumour invasion, extensive *in vitro* data showed that CA IX inhibition may affect tumour cell survival and proliferation^27^ and/or motility and invasion^28^, dependently on the tumour cell type. In this work we focussed only on the proliferative outcome of CA IX inhibition in order to evaluate the effect of the membrane-impermeant compound C18 in comparison with the more permeable derivatives SLC-0111 and AAZ, showing a rather different anti-proliferative outcome. Finally, as already reported for other tumour types^3^^,^^4^^,^^27–31^, it will be extremely interesting to validate the efficacy of these compounds in combination with conventional and/or new chemotherapeutic treatments in order to assess if CA inhibition may improve the response or prevent the onset of resistance to these therapies.
